# Is It Benign or Is It a Pariah? Empirical Evidence for the Impact of the Common Myna (*Acridotheres tristis*) on Australian Birds

**DOI:** 10.1371/journal.pone.0040622

**Published:** 2012-07-11

**Authors:** Kate Grarock, Christopher R. Tidemann, Jeffrey Wood, David B. Lindenmayer

**Affiliations:** 1 Fenner School of Environment and Society (College of Medicine, Biology and Environment), The Australian National University, Canberra, Australian Capital Territory, Australia; 2 Invasive Animals Cooperative Research Centre, University of Canberra, Australian Capital Territory, Australia; Institut Pluridisciplinaire Hubert Curien, France

## Abstract

There is widespread concern over the impact of introduced species on biodiversity, but the magnitude of these impacts can be variable. Understanding the impact of an introduced species is essential for effective management. However, empirical evidence of the impact of an introduced species can be difficult to obtain, especially when the impact is through competition. Change in species abundance is often slow and gradual, coinciding with environmental change. As a result, negative impacts on native species through competition are poorly documented. An example of the difficulties associated with obtaining empirical evidence of impact due to competition comes from work on the Common Myna (*Acridotheres tristis*). The species is listed in the World’s top 100 worst invaders, despite a lack of empirical evidence of its negative impacts on native species. We assessed the impact of the Common Myna on native bird abundance, using long-term data both pre and post its invasion. At the outset of our investigation, we postulated that Common Myna establishment would negatively affect the abundance of other cavity-nesting species and bird species that are smaller than it. We found a negative relationship between the establishment of the Common Myna and the long-term abundance of three cavity-nesting species (Sulphur-crested Cockatoo, Crimson Rosella, Laughing Kookaburra) and eight small bird species (Striated Paradoxes, Rufous Whistler, Willie Wagtail, Grey Fantail, Magpie-lark, House Sparrow, Silvereye, Common Blackbird). To the best of our knowledge, this finding has never previously been demonstrated at the population level. We discuss the key elements of our success in finding empirical evidence of a species impact and the implications for prioritisation of introduced species for management. Specifically, prioritization of the Common Myna for management over other species still remains a contentious issue.

## Introduction

There is widespread concern over the impact of introduced species on biodiversity [1], [2] and the number of these introductions is increasing globally [3], [4]. Introduced species can affect native species through competition, predation, herbivory, habitat alteration, disease or hybridization [5], [6], [7]. These impacts can lead to changes in population dynamics of native species, altered community structure, and altered ecosystem services [5], [8], [9], [10], [11].

The magnitude of impacts of an introduced species can be variable. Some have a devastating impact while others are relatively benign [12], [13]. For example, the invasive marine alga *Caulerpa taxifolia* spread rapidly throughout the Mediterranean Sea, with devastating impacts on other algal species, seagrasses and sessile invertebrates [14]. Conversely, Davis *at al.* [12] describes the long-term eradication program of the tamarisk shrub (*Tamarix spp.*). This species was introduced to the United States in the 1930s and its management currently costs an estimated $US 80 million annually. The impact of tamarisk is poorly understood and evidence suggests it assists riverbank stabilisation and provides nesting sites for threatened native birds [12].

Understanding the impact of an introduced species is essential for effective management [15]. Due to limited resources, management prioritization should be given to introduced species that have the greatest undesirable impact [12], [15]. The traditional belief that all introduced species have a negative impact can lead to wasteful allocation of resources (see tamarisk shrub example above) [12]. Understanding a species impact facilitates targeted management to ameliorate impacts [12], [15], [16], [17], [18], [19].

Empirical evidence of the impact of an introduced species can be very difficult to obtain for three key reasons:

1. A lack of long-term data prior to, and then after, invasion [20];
2. Environmental change occurring alongside species introductions, making it hard to distinguish species impacts from the impacts of environmental change (eg habitat clearing, climate change) [20], [21], [22]; and
3. A poor understanding of the mechanisms of impact (eg competition vs. predation) [18], [23], [24].

Particular difficulties arise when trying to obtain empirical evidence of impact due to competition [20]. This is because changes in species abundance due to competition may be slow [6], [25], and frequently occur in combination with other environmental impacts (eg native habitat clearing) [21], [22]. The impacts of competition often occur more slowly than, for example, predation where a predator immediately kills their prey [6]. Observations of negative encounters between species are useful for determining the mechanisms of impact [18], [23], [24]. However, long-term changes in the abundance of affected species provide much stronger evidence of impact and competition [26].

A good example of the difficulties associated with obtaining empirical evidence of impact due to competition is the Common Myna (*Acridotheres tristis*). Concern has been raised that the Common Myna affects native birds in three ways: (1) competition for food; (2) competition for cavity-nesting sites; and (3) competition for territories [27], [28], [29], [30]. Research from around the world has investigated competition between the Common Myna and native bird species [28], [31]. However, to the best of our knowledge, no study to date has provided empirical evidence of the species impact on the long-term abundance of native bird species (see *Study Species* in the Methods section).

The Common Myna is listed in the top 100 of the world’s worst invaders, despite a lack of empirical evidence of negative impacts on native species [32]. Is the lack of evidence for Common Myna impact due to the difficulty in obtaining evidence of impact (especially due to competition)? Or, has the Common Myna fallen victim to the traditional belief that all introduced species have a negative impact [12]?

In this paper, we assess the impact of Common Myna establishment on long-term bird abundance. We investigated the abundance of 20 bird species in Canberra in south-east Australia, pre and post Common Myna establishment. These bird species included seven cavity-nesting, ten small (<25 cm head to tail) and five large (>30 cm head to tail) species of bird (Table1,2,3).

**Table 1:** Cavity-nesting species autoregressive analysis^1,2,3^.

Species	AR1 phi_1 (estimate)	Season non-breeding	Dwellingsper km	Population density per km	Native grassland	Dry forest	Modified urban grassland	Woodland	Tree cover	Year	Years after Common Myna establishment
Galah (*Cacatua roseicapilla*)	0.47±0.05					10.42±2.57 p<0.001	6.73±1.22 p<0.001			3.91±1.23 p = 0.002	−1.41±1.30 p = 0.284
**Sulphur-crested Cockatoo (** ***Cacatua galerita*** **)**	**0.20±0.05**	−**27.59±5.45 p<0.001**							**2.11±0.39 p<0.001**	**10.31±0.78 p<0.001**	−**1.97±0.75 p = 0.010**
Australian King-parrot (*Alisterus scapularis*)	0.76±0.04	−5.73±0.94 p<0.001					2.42±0.83 p = 0.006		2.74±0.51 p<0.001	0.79±0.63 p = 0.224	2.33±0.62 p<0.001
**Crimson Rosella (** ***Platycercus elegans*** **)**	**0.59±0.05**	−**12.90±0.88 p<0.001**							**2.35±0.15 p<0.001**	**5.86±0.30 p<0.001**	−**3.45±0.30 p<0.001**
Eastern Rosella (*Platycercus eximius*)	0.55±0.05							9.03±1.80 p<0.001		−0.71±0.23 p = 0.003	1.10±0.25 p<0.001
**Laughing Kookaburra (** ***Dacelo novaeguineae*** **)**	**0.69±0.05**						−**0.79±0.21 p<0.001**	−**5.07±2.25 p = 0.030**		**0.02±0.15 p = 0.884**	−**0.39±0.18 p = 0.030**
Common Starling (*Sturnus vulgaris*)	0.30±0.05				−7.36±3.19 p = 0.024				−3.55±0.80 p<0.001	−17.35±1.58 p<0.001	2.00±1.52 p = 0.195

1 Species abundance that has a negative relationship with Common Myna establishment are highlighted in bold font.

2 Autoregressive models of order one (AR1) are reported in the table.

3 The Common Starling is an introduced species in Australia.

**Table 2:** Small bird species (<25 cm head to tail) autoregressive analysis^1,2,3^.

Species	AR1 phi_1 (estimate)	Season non-breeding	Dwellings per km	Population density per km	Native grassland	Dry forest	Modified urban grassland	Woodland	Tree cover	Year	Years after Common Myna establishment
**Superb Fairy-wren** **(** ***Malurus cyaneus*** **)**	**0.42±0.06**	−**6.56±0.82 p<0.001**				−**1.34±0.43 p = 0.003**	−**1.81±0.20 p<0.001**			**1.77±0.21 p<0.001**	−**0.89±0.22 p<0.001**
**Striated Pardalote (** ***Pardalotus striatus*** **)**	**0.39±0.06**	**1.68±0.56 p = 0.003**	−**0.06±0.01 p<0.001**							**0.51±0.12 p<0.001**	−**0.70±0.12 p<0.001**
**Willie Wagtail (** ***Rhipidura leucophrys*** **)**	**0.53±0.05**	−**1.62±0.37 p<0.001**		−**0.02±0.01 p = 0.011**				−**4.21±1.78 p = 0.022**	−**0.25±0.08 p = 0.004**	**0.17±0.20 p = 0.417**	−**0.77±0.21 p<0.001**
**Grey Fantail (** ***Rhipidura fuliginosa*** **)**	**0.29±0.06**	−**1.65±0.38 p<0.001**					**1.23±0.12 p<0.001**		**0.80±0.08 p<0.001**	**0.82±0.09 p<0.001**	−**0.91±0.09 p<0.001**
**Magpie Lark (** ***Grallina cyanoleuca*** **)**	**0.29±0.06**			−**0.09±0.02 p<0.001**			**3.43±0.55 p<0.001**		**1.05±0.20 p<0.001**	**2.67±0.35 p<0.001**	−**2.24±0.32 p<0.001**
House Sparrow (*Passer domesticus*)	0.73±0.05	11.71±3.22 p<0.001	0.90±0.18 p<0.001		−10.53±4.13 p = 0.019					−6.62±1.70 p<0.001	−1.59±1.66 p = 0.348
**Silvereye** **(** ***Zosterops lateralis*** **)**	**0.43±0.06**	**9.63±2.48 p<0.001**					**3.90±1.04 p<0.001**		**4.26±0.65 p<0.001**	**0.45±0.77 p = 0.560**	−**2.65±0.75 p<0.001**
**Common Blackbird** **(** ***Turdus merula*** **)**	**0.91±0.03**						**3.25±0.89 p = 0.003**		**1.89±0.50 p<0.001**	**2.83±0.48 p<0.001**	−**2.78±0.50 p<0.001**

1 Species abundance that has a negative relationship with Common Myna establishment are highlighted in bold font.

2 Autoregressive models of order one (AR1) are reported in the table.

3 The House Sparrow and Common Blackbird are introduced species in Australia.

**Table 3:** Large bird species (>30 cm head to tail) autoregressive analysis^1^.

Species	AR1 phi_1 (estimate)	Season non-breeding	Dwellings per km	Population densityper km	Native grassland	Dry forest	Modifiedurbangrassland	Woodland	Tree cover	Year	Years after Common Myna establishment
Red Wattlebird (*Anthochaera carunculata*)	0.23±0.06			0.05±0.01 p<0.001		−3.48±1.19 p = 0.005		25.27±7.91 p = 0.002	0.76±0.19 p<0.001	1.27±0.40 p = 0.002	0.95±0.44 p = 0.033
Noisy Friarbird (*Philemon corniculatus*)	0.34±0.06	−2.61±0.74 p<0.001						6.49±1.29 p<0.001		−0.90±0.17 p<0.001	0.13±0.19 p = 0.487
Australian Magpie(*Gymnorhina tibicen*)	0.55±0.06	4.24±0.89 p<0.001								1.15±0.27 p<0.001	0.34±0.27 p = 0.217
Pied Currawong (*Strepera graculina*)	0.17±0.05	7.90±3.15 p = 0.013	0.35±0.10 p<0.001				−4.71±0.84 p<0.001			−0.98±0.64 p = 0.129	1.23±0.55 p = 0.028
Australian Raven (*Corvus coronoides*)	0.58±0.05	0.86±0.42 p = 0.041		0.02±0.01 p = 0.014	−1.94±0.38 p<0.001				0.38±0.13 p = 0.005	0.66±0.15 p<0.001	0.15±0.14 p = 0.310

1 Autoregressive models of order one (AR1) are reported in the table.

Earlier studies indicated that the Common Myna may affect cavity-nesting species through competition for nest sites, reducing the breeding success of these species [29], [30]. Therefore, at the outset of our investigation, we postulated that Common Myna establishment would negatively affect the abundance of cavity-nesting species. Earlier studies also indicated that the Common Myna is a territorial species actively defending an area of one to three hectares (see [31] for a review). Territorial exclusion by another species of bird, the Noisy Miner (*Manorina melanocephala*), an Australian native species, is known to primarily affect other small insectivorous bird species [33] and research on the Common Myna suggests a similar pattern [27]. Therefore, we postulated that Common Myna establishment would negatively affect the abundance of small bird species, but not large bird species. We discuss the implications of our findings and the complexities associated with the management prioritisation of one species over another.

## Materials and Methods

### Study Species

The Common Myna is from the Sturnidae family and is a sedentary bird measuring 23–25 cm in length [28]. The species is a highly adaptable generalist omnivore, foraging within 1–3 km of a communal roost [34], [35]. The Common Myna forms lifelong monogamous breeding pairs that aggressively defend the same territory each nesting season [36], [37]. The species is primarily a cavity-nesting species throughout its introduced range, laying between two to seven eggs per clutch [28], [31]. The Common Myna thrives in human-modified environments, reaching high densities of more than 200 birds per km^2^ in cities and towns [31]. The species is also found along roadsides, in coastal mangroves, and in open forest habitats [28]. The Common Myna tends to avoid dense forest but landscape fragmentation can lead to increases in its abundance [34].

The Common Myna originates from India and central and southern Asia [28], [39]. It has been introduced all over the world and has become established on all continents except Antarctica [28]. The species was introduced primarily to control insect pests in agriculture [28], [34], [38].

The Common Myna [39] was first brought to Australia in 1862 to control insects in market gardens in the city of Melbourne [40]. The species quickly established in Melbourne and that population became a source population for other introductions within Australia [40]. The Common Myna is now well established in many cities and towns along the east coast of Australia [41].

The first published record of the Common Myna in our study area of Canberra (a city of 370 000 people) was of a pair of birds in 1968 [42]. Since then, Common Myna numbers in Canberra have steadily increased [43].

### Long-term Data

We used long-term survey data gathered by the Canberra Ornithologists Group (COG) to document bird abundance over 29 years in Canberra. COG established the Canberra Garden Bird Survey (GBS) in 1981 (COG, 2010). The GBS volunteers survey birds in and around the city of Canberra. Observers survey an area of 3.1 ha. Surveys are conducted fortnightly for a 20-minute period. A total of 74 492 surveys was undertaken in the survey area over 29 years. Further detail on survey procedures are provided by [43].

### Target Species Abundance Analysis

**Figure 1:**
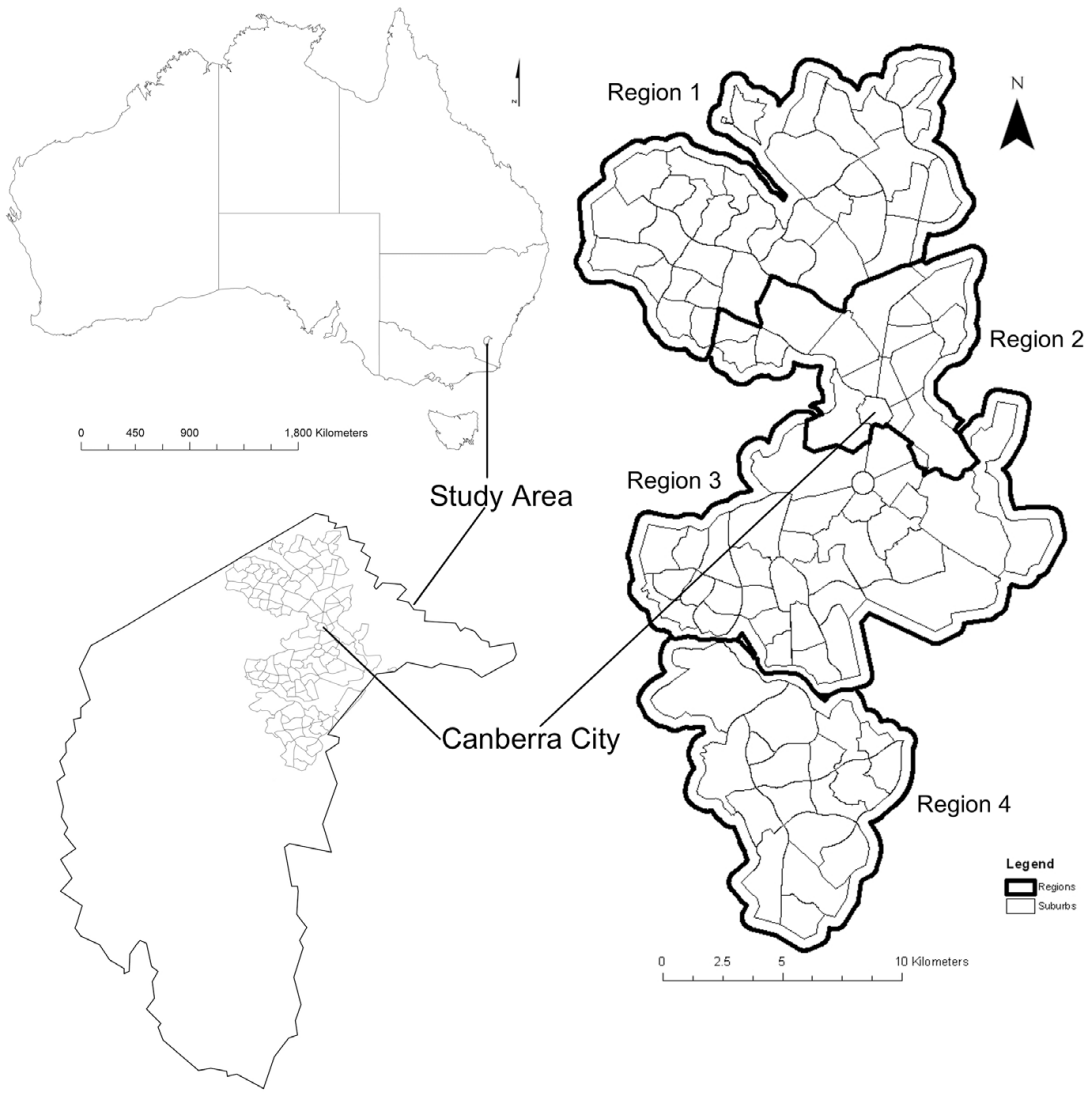
Location of the four Regions in Canberra, South East Australia.

We determined the abundance of 20 bird species in Canberra over 29 years using data from the COG GBS, comprising seven cavity-nesting (Table 1), eight small species (<25 cm head to tail) (Table 2) and five large species (>30 cm head to tail)(Table 3). We used ArcGIS 10® [44] to define four geographic regions in Canberra (Figure 1). This enabled grouping of survey sites to ensure continuity of survey effort over each region and year. We based regions primarily on geographic location and development history of the city. There was a total of 352 survey sites, with 71 in Region 1, 107 in Region 2, 133 in Region 3 and 41 in Region 4. The mean number of surveys undertaken each year was 496±33 in Region 1, 873±28 in Region 2, 898±32 in Region 3 and 259±15 in Region 4.

**Figure 2:**
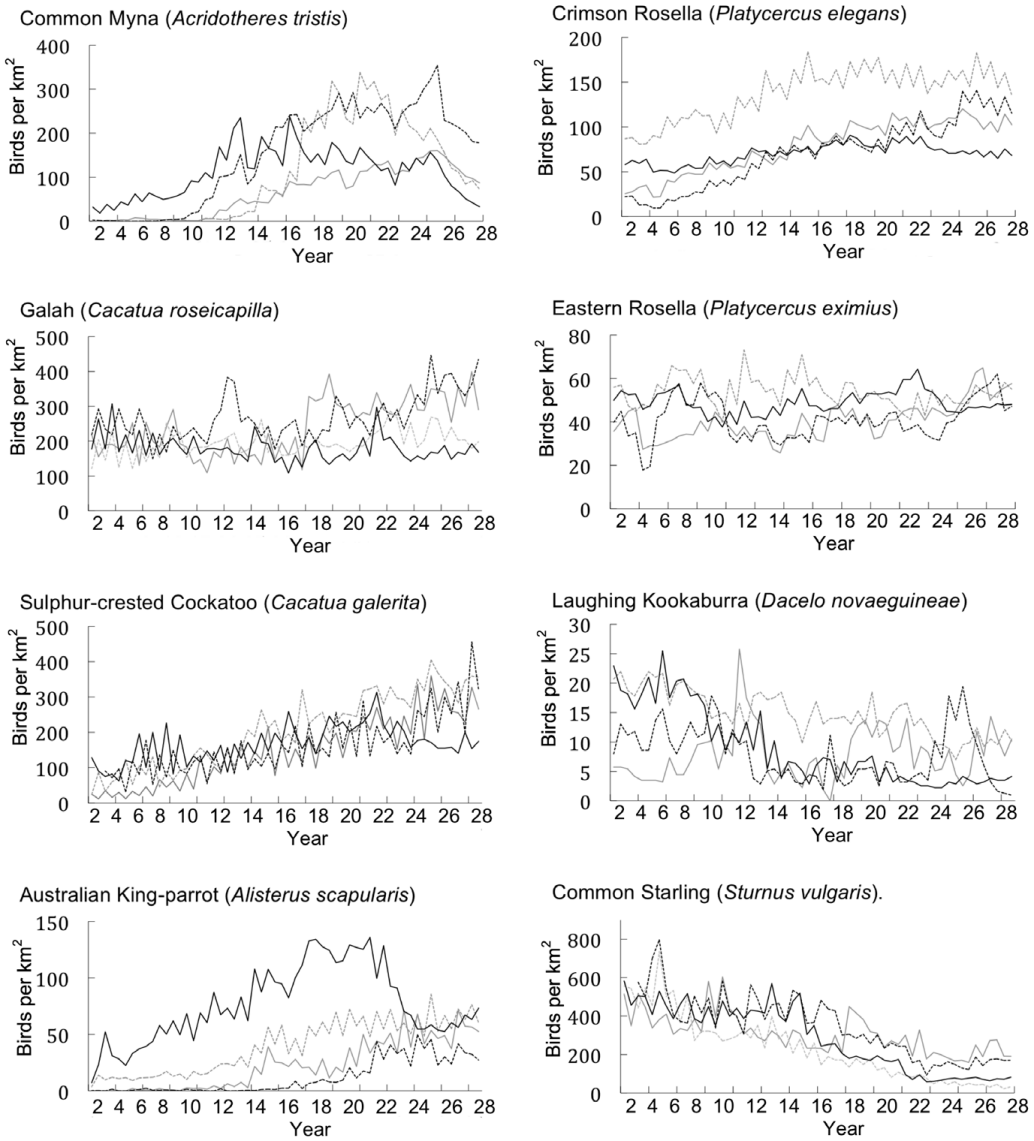
The bi-annual abundance (birds per km^2^) of cavity-nesting species across four Regions in the rural city of Canberra, South East Australia (Region 1: solid grey line, Region 2: dotted grey line, Region 3: solid black line, Region 4: dotted black line).

**Figure 3:**
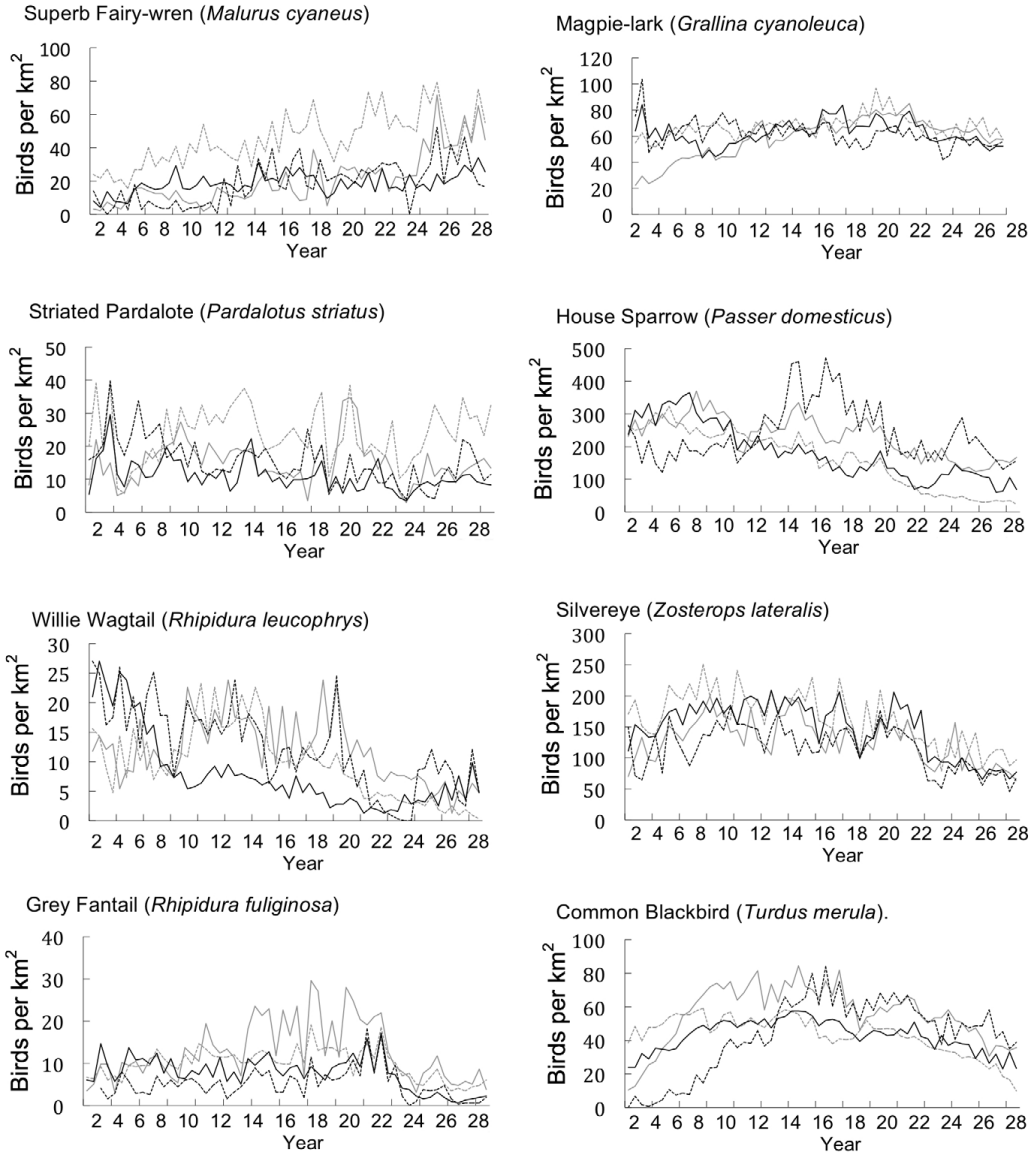
The bi-annual abundance (birds per km^2^) of small bird species (<25 cm head to tail) across four regions in the rural city of Canberra, South East Australia (Region 1: solid grey line, Region 2: dotted grey line, Region 3: solid black line, Region 4: dotted black line).

**Figure 4:**
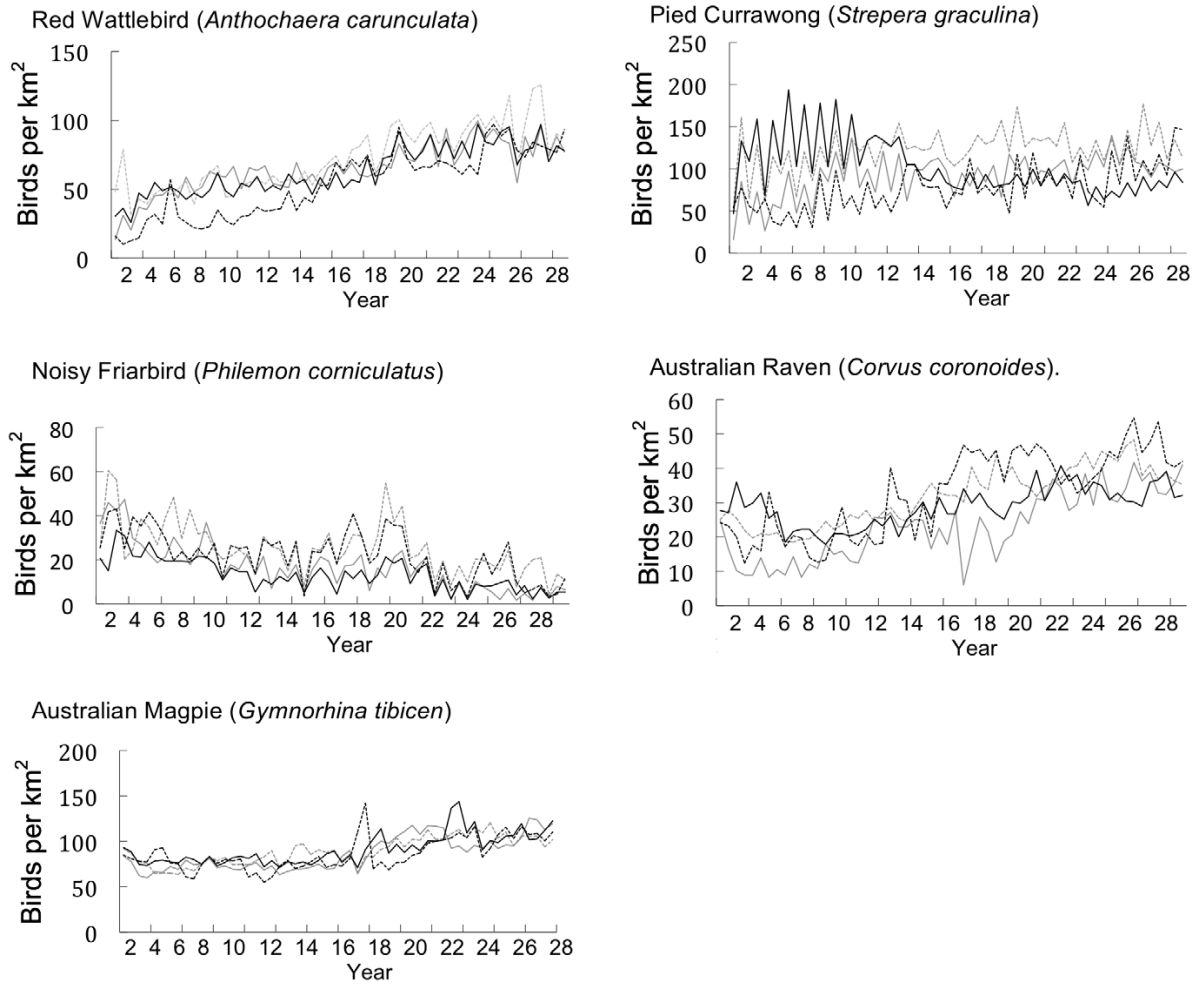
The bi-annual abundance (birds per km^2^) of large bird species (>30 cm head to tail) across four regions in the rural city of Canberra, South East Australia (Region 1: solid grey line, Region 2: dotted grey line, Region 3: solid black line, Region 4: dotted black line).

We split years into two seasons: breeding (September to February) and non-breeding seasons (March to August). For each species, we used GenStat 14® [45] to fit hierarchical generalized linear models [46] to raw counts of individuals using a quasi-Poisson model with a logarithmic link function. We treated region, year, season, and their interactions as fixed effects. We treated sites as a random effect with a log-gamma distribution. For each combination of region, year and season, we estimated the average number of birds per site, thus reducing the data from the results of 74 492 individual surveys to 232 estimates. Each site covered an area of 3.142 ha, and we converted the estimates to abundance per km^2^ by multiplying by (100/3.142). Using this method, we calculated the bi-annual abundance of each target species per km^2^ per region and graphed the results (Figure 2, 3, 4).

### Target Species Autoregressive Analysis

We fitted autoregressive models in GenStat 14® [45] to investigate the impact of the Common Myna on our 20 target species (Figure 2). We fitted autoregressive models for each of our 20 target species using their bi-annual abundance as the response variable in the model. The candidate fixed variables included in the modeling were season, urban development (dwellings per km^2^, population per km^2^) and vegetation type (native grassland, dry forest, modified urban grassland, woodland, tree cover). We also included the fixed variables of year, and years after Common Myna establishment, in the model. The random model was set to region and time and we used autoregressive models of order one (AR1) and two (AR2), and Wald tests for dropping individual terms from the full fixed model. We sequentially removed the least significant explanatory variable from the model, continuing this process until only significant (<0.05) explanatory variables remained (with the exception of the variables year and years after Common Myna establishment, which were included in all models) (Table 1, 2, 3). We used a table of effects to predict the impact of each significant variable (±SE).

We defined Common Myna establishment for each region to have occurred when there was an estimated mean of two (±SE) individuals of the Common Myna per km^2^. The years after Common Myna establishment had a zero value in the years proceeding (and including the year of) Common Myna establishment. The years after Common Myna establishment were numbered sequentially. We used establishment of the Common Myna rather than Common Myna density to investigate impact as this enabled us to investigate species abundance prior to, and following, the presence of the Common Myna in Canberra. The Common Myna has the potential to affect the abundance of other species even when it occurs at low densities. The Common Myna exhibits territorial behavior, feeds within an area of up to two km from a communal roost [28], [31], [35], [36], [37], and builds multiple nests that may deter other cavity-nesting species [29], [30].

We sourced data on the number of dwellings and human population density from the Australian Bureau of Statistics [47]. Data were available for six intervals throughout the survey period for each suburb in Canberra (1981, 1986, 1991, 1996, 2001, 2006). We determined the average dwelling density and human population density by calculating the mean of all suburbs within each region. We calculated vegetation variables (native grassland, dry forest, modified urban grassland, woodland) using ArcGIS layer from the ACT Department of Lands [48]. Numerous features were available on the ArcGIS layer from the ACT Department of Lands (e.g ‘urban area’). However, where possible, we used data that were updated throughout the survey period. For example, we used data from the Australian Bureau of Statistics on dwelling density (updated six times over the 29 year period) instead of ‘urban area’ from the ArcGIS layer from the ACT Department of Lands. We also determined percent tree cover from Landsat satellite imagery updated 11 times throughout the survey period (1995, 1998, 2000, 2002, 2004, 2005, 2006, 2007, 2008, 2009, 2010) [49].

For each of our 20 target species, we compared the AR1 with AR2 models to ensure estimates for years after Common Myna establishment reported similar values (eg a positive or negative impact, of a similar magnitude). The AR1 model compares a species abundance with the previous seasons abundance. More specifically, it looks at the relationship between a species breeding season abundance and the preceding non-breeding season abundance. The AR2 model compares a species abundance with the previous season of the same type. For example it compares the abundance of a species in the breeding season to the abundance of the species in the breading season one year prior. We report AR1 models throughout our results for all species, after comparison between the two models revealed that estimates for Common Myna impact were consistent between models and the AR2 coefficients were rarely significant.

We were able to distinguish between the impacts of the Common Myna and other causal factors through the inclusion of urban development and vegetation variables. More specifically, we could avoid incorrectly identifying the Common Myna as having an impact if a common-causal variable was responsible for the decline. For example, urban development may negatively affect some species [50], and attributing that negative impact to the Common Myna would be erroneous.

## Results

Our analysis of GBS records indicated that the Common Myna became established in Region 1 in 1991, Region 2 in 1993 and Region 4 in 1989. The Common Myna was already established in Region 3 prior to the commencement of GBS surveys. After establishment, the abundance of the Common Myna increased each year by an estimated 6.4 (±2.5) birds per km^2^ each year (F_1,40_ = 6.6, P = 0.014). On average, the abundance of the Common Myna increased throughout the survey period by an estimated 0.8 (±2.4) birds per km^2^ each year although this was not statistically significant (F_1,57_ = 0.1, P = 0.723). We found significant positive relationships between the abundance of the Common Myna and dry forest (F_1,12_ = 10.8, P = 0.007), modified grassland (F_1,26_ = 7.6, P = 0.010), and tree cover (F_1,75_ = 6.7, P = 0.012).

### Impacts on Cavity-nesting Species

We found a significant negative relationship between the establishment of the Common Myna and the abundance of the Sulphur-crested Cockatoo (F_1,77_ = 6.9, P = 0.010), the Crimson Rosella (F_1,33_ = 135, P<0.001) and the Laughing Kookaburra (F_1,52_ = 5.0, P = 0.030). Sulphur-crested Cockatoo abundance increased throughout the survey period by an estimated 10.3 (±0.8) birds per km^2^ each year. However, after Common Myna establishment, growth in abundance reduced by an estimated 2.0 (±0.7) birds per km^2^ each year. Crimson Rosella abundance increased throughout the survey period by an estimated 5.9 (±0.3) birds per km^2^ each year. However, after Common Myna establishment, growth in abundance declined by an estimated 3.5 (±0.3) birds per km^2^ each year. Laughing Kookaburra abundance was relatively stable throughout the survey period. However, after Common Myna establishment, abundance reduced by an estimated 0.4 (±0.2) birds per km^2^ each year.

We found no significant negative relationships between Common Myna establishment and the abundance of the Galah, Australian King-Parrot, Eastern Rosella or Common Starling. The abundance of the Galah increased over the 29-year study with growth in abundance declining after Common Myna establishment, but this change was not statistically significant (F_1,52_ = 1.2, P = 0.284) (Table 1). The abundance of the Australian King-Parrot, Eastern Rosella and Common Starling appeared to increase after Common Myna establishment (Table 1, Figure 2).

### Impacts on Small Bird Species

We found a significant negative relationship between Common Myna establishment and the abundance of seven of the eight small bird species we examined (Table 2). The abundance of the Superb Fairy-wren, Striated Pardalote, Willie Wagtail, Grey Fantail, Magpie Lark, Silvereye and Common Blackbird increased throughout the survey period (Table 2, Figure 3). However, after Common Myna establishment, growth in abundance of these bird species declined significantly (Table 2). House Sparrow abundance declined throughout the survey period by an estimated 6.6 (±1.7) birds per km^2^ each year. After Common Myna establishment, abundance continued to decline by an estimated 1.6 (±1.7) birds per km^2^ each year, although this was not statistically significant (F_1,20_ = 0.9, P = 0.348)(Table 2).

### Impacts on Large Species

We found no negative relationships between Common Myna establishment and the abundance of all of the five large bird species we analysed: Red Wattlebird, Noisy Friarbird, Australian Magpie, Pied Currawong and Australian Raven (Table 3). Red Wattlebird, Australian Magpie and Australian Raven abundance increased over 29 years (Table 3). Noisy Friarbird abundance declined over the study period by an estimated 0.9 (±0.2) birds per km^2^ each year (F_1,50_ = 85.7, P<0.001). Pied Currawong abundance did not differ significantly over the study period (Table 3).

## Discussion

Previous attempts to investigate Common Myna impact have relied on short-term data (from one to three years) [27], [29], [51] with limited success. Our long-term data and integrated approach provided a unique opportunity to present the strongest evidence to date for the impact of the Common Myna on native bird species. Incorporating variables for environmental change in our model enabled us to obtain a better understanding of the impact of Common Myna establishment on bird abundance. Our model was designed to incorporate changes in species abundance due to habitat modification, thus enabling an understanding of the impact of the Common Myna in a changing environment.

Our analysis suggests that the Common Myna had a negative impact on the long-term abundance of some cavity-nesting bird species and some small bird species. These species include Sulphur-crested Cockatoo, Crimson Rosella, Laughing Kookaburra, Superb Fairy-wren, Striated Pardalote, Willie Wagtail, Grey Fantail, Magpie-lark, Silvereye and Common Blackbird. To the best of our knowledge, this finding for the Common Myna has never previously been demonstrated at the population level.

### Cavity-nesting Species

At the outset of this study, we postulated that Common Myna establishment would negatively affect the abundance of cavity-nesting species. This was supported by our data for the Sulphur-crested Cockatoo, Crimson Rosella and Laughing Kookaburra (Table 1). It was not supported by our data for the Galah, Australian King-Parrot, Eastern Rosella or Common Starling (Table 1). The negative impact of Common Myna establishment on Crimson Rosella abundance is consistent with a previous study that quantified nest-cavity competition between these two species [29]. The negative impact of Common Myna establishment on the Sulphur-crested Cockatoo and the Laughing Kookaburra is especially interesting as they are larger than the Common Myna (44–51 cm and 41–47 cm respectively). However, anecdotal evidence suggests that the Common Myna is capable of displacing large bird species and even mammals from cavity-nesting sites [52].

We found no significant negative relationships between Common Myna establishment and Common Starling abundance. This finding was unexpected, as several studies have observed intense nest-cavity competition between these two species, concluding that the Common Myna is responsible for a decline in Common Starling numbers [27], [37]. Common Starling abundance declined throughout the survey period (Figure 2). Declining Common Starling numbers also have been reported in South-eastern Australia [53]. The declining abundance of the Common Starling may have reduced our ability to detect an impact from the Common Myna.

### Small Bird Species (<25 cm)

Our postulate that Common Myna establishment would negatively affect the abundance of small bird species was supported by our data for the Superb Fairy-wren, Striated Pardalote, Willie Wagtail, Grey Fantail, Magpie-lark, Silvereye and Common Blackbird. This result was broadly consistent with earlier studies reporting that the Common Myna aggressively forces birds out of an area [27], [31], [37].

### Large Bird Species (>30 cm)

Our postulate that Common Myna establishment would not negatively affect the abundance of large bird species was supported for all of the species we analysed including the Red Wattlebird, Noisy Friarbird, Australian Magpie, Pied Currawong and Australian Raven.

### Is it Benign or is it a Pariah? Implications for Management

Our results highlighted the extent to which the Common Myna influences both cavity-nesting and small bird species. We conclude that the effect of the Common Myna on native bird species in the Canberra area is not benign. However, there are still questions regarding the seriousness of this impact and the type of management (if any) that is warranted. In Sydney, the Common Myna is believed to have little impact on native bird species, with anthropogenic habitat modification believed to be the main driver of native species decline [51], [54]. Due to limited resources for management and increasing numbers and types of introduced species, the appropriate management response for the Common Myna remains a contentious issue.

The bird species we investigated in this study were neither rare nor threatened (three are introduced). These mechanisms of impact (competition for nest-cavities and territory) also may influence threatened species. However, we were unable to demonstrate the impact of the Common Myna on threatened species such as the Superb Parrot (*Polytelis swainsonii*) because limited observations of such species.

Regardless of its impact, the Common Myna is considered by the public to be a pariah. In Australia in 2005, the species was voted as the ‘most significant pest’, ‘the pest problem seen to be increasing most’ and the top ‘pest problem that needs more control’ [55]. Community concern about the Common Myna was greater than devastating species such as the Cane Toad (*Rhinella marina*), Red Fox (*Vulpes vulpes*), Feral Cat (*Felis catus*) and European Rabbit (*Oryctolagus cuniculus*). Perhaps this is partly due to the Common Myna being abundant and visible in urban areas. Although this community passion for Common Myna management is positive, we must not let it cloud rational scientific judgment and the strategic allocation of pest management resources [12], [15], [16], [17], [18], [19].

In Australia, native birds are also negatively affected by two native bird species, the Noisy Miner (Manorina *melanocephala*) and Bell Miner (Manorina *melanophrys*) [33]. Research suggests that land use practices, such as habitat clearing can lead to increases in Noisy Miner and Bell Miner abundance, which then territorially exclude native bird species [33], [56], [57], [58], [59], [60]. Some researchers suggest Noisy Miner populations should be culled in certain areas of Australia [61]. Prioritisation of management must not be influenced by the origins of the species [12].

### Prioritization of Introduced Species Management

Understanding a species impact is vital for effective management and the prioritization of limited resources [12], [15]. Prioritization of introduced species management has been recognised by numerous studies that attempt to rank the impacts of introduced species [62], [63], [64], [65]. Such studies have focused on plants [63], mammals [62], and more recently bird species [64], [65]. Debate over the accuracy of prioritization assessments exists primarily due to a lack of scientific evidence for species impact [66], [67]. Lack of evidence for a species impact causes risk assessments to be based on hypotheses or anecdotal observations of impact, creating significant variation between assessments (eg [65] vs. [64]).

As outlined in this paper, empirical evidence of the impact of introduced species can be difficult to obtain. As a result, impacts of introduced species are poorly documented, especially when the impact is through competition [6]. For example, a review of the impacts of introduced bird species concluded that there is currently little evidence that introduced birds strongly influence native species through competition or predation [68]. More recently, an assessment of the impact of introduced bird species in Europe concluded that knowledge on the ecology and impact of introduced birds was poor [65].

Different mechanisms of introduced species impact further add to the complexity management prioritization. The impacts of a predator can be severe and immediate, especially when compared to competition that can take many years to affect species abundance, as seen in our study [6], [7], [69]. Due to limited resources and short funding cycles, management prioritization may focus on a species with clear and immediate impacts, rather than a species that slowly reduce the abundance of a native species.

The difficulties associated with the prioritization of species management highlight the importance of studies like ours that attempt to obtain empirical evidence of a species impact. Our case study on the Common Myna provides us with six key findings. These being:

1. Long-term datasets pre and post species invasion provide important resources for evaluating species impact.
2. Incorporating environmental change into species impact analysis is essential to enable discrimination between the species impact and other forms of impact (eg habitat clearing).
3. There is a heightened difficulty of documenting impact on species with low or fluctuating abundance due to limited data and thus a reduced ability to detect temporal shifts in their abundance.
4. Prior scientific observations on the mechanisms of species impact are essential to provide firm reasoning for observed changes in native species abundance.
5. Empirical evidence of a species’ impact is critical for the prioritisation of introduced species management.
6. Even with empirical evidence of a specie’s impact, the prioritisation of introduced species management may remain a contentious issue, due to variability in impact severity and the different time periods over which impacts occur.
